# Human pegivirus-1 replication influences NK cell reconstitution after allogeneic haematopoietic stem cell transplantation

**DOI:** 10.3389/fimmu.2022.1060886

**Published:** 2023-01-11

**Authors:** Amandine Pradier, Samuel Cordey, Marie-Céline Zanella, Astrid Melotti, Sisi Wang, Anne-Claire Mamez, Yves Chalandon, Stavroula Masouridi-Levrat, Laurent Kaiser, Federico Simonetta, Diem-Lan Vu

**Affiliations:** ^1^ Faculty of Medicine, University of Geneva, Geneva, Switzerland; ^2^ Division of Haematology, Department of Oncology, Geneva University Hospitals, Geneva, Switzerland; ^3^ Translational Research Center for Oncohematology, Department of Medicine and Department of Pathology and Immunology, Faculty of Medicine, University of Geneva, Geneva, Switzerland; ^4^ Laboratory of virology, Division of Laboratory Medicine, Geneva University Hospitals, Geneva, Switzerland; ^5^ Division of Infectious diseases, Geneva University Hospitals, Geneva, Switzerland; ^6^ Center for emerging viruses, Geneva University Hospitals, Geneva, Switzerland

**Keywords:** human pegivirus-1, NK cell, transplantation, stem cell, CD16, granzyme B, CD57

## Abstract

**Introduction:**

Human pegivirus-1 (HPgV-1) is a so-called commensal virus for which no known associated organ disease has been found to date. Yet, it affects immune-reconstitution as previously studied in the HIV population, in whom active co-infection with HPgV-1 can modulate T and NK cell activation and differentiation leading to a protective effect against the evolution of the disease. Little is known on the effect of HPgV-1 on immune-reconstitution in allogeneic hematopoietic stem cell transplant (allo-HSCT) recipients, a patient population in which we and others have previously reported high prevalence of HPgV-1 replication. The aim of this study was to compare the immune reconstitution after allo-HSCT among HPgV-1-viremic and HPgV-1-non-viremic patients.

**Methods:**

Within a cohort study of 40 allo-HSCT patients, 20 allo-HSCT recipients positive in plasma sample for HPgV-1 by rRT-PCR during the first year (1, 3, 6, 12 months) after transplantation were matched with 20 allo-HSCT recipients negative for HPgV-1. T and NK cell reconstitution was monitored by flow cytometry in peripheral blood samples from allo-HSCT recipients at the same time points.

**Results:**

We observed no significant difference in the absolute number and subsets proportions of CD4 and CD8 T cells between patient groups at any analysed timepoint. We observed a significantly higher absolute number of NK cells at 3 months among HPgV-1-viremic patients. Immunophenotypic analysis showed a significantly higher proportion of CD56^bright^ NK cells mirrored by a reduced percentage of CD56^dim^ NK cells in HPgV-1-positive patients during the first 6 months after allo-HSCT. At 6 months post-allo-HSCT, NK cell phenotype significantly differed depending on HPgV-1, HPgV-1-viremic patients displaying NK cells with lower CD16 and CD57 expression compared with HPgV-1-negative patients. In accordance with their less differentiated phenotype, we detected a significantly reduced expression of granzyme B in NK cells in HPgV-1-viremic patients at 6 months.

**Discussion:**

Our study shows that HPgV-1-viremic allo-HSCT recipients displayed an impaired NK cell, but not T cell, immune-reconstitution compared with HPgV-1-non-viremic patients, revealing for the first time a potential association between replication of the non-pathogenic HPgV-1 virus and immunomodulation after allo-HSCT.

## Introduction

Commensal viruses composing the human virome are silently replicating in the host without inducing known disease but interacting with other components of the microbiome as well as the host immune system (1, 2). Human pegivirus-1 (HPgV-1) is a so-called commensal virus for which no known associated organ disease has been found to date. Yet, in HIV-infected individuals it was reported that active co-infection with HPgV-1 can provide a protective effect against evolution to AIDS and mortality (3–5). HPgV-1 seems to inhibit lymphocyte differentiation and exhaustion, which could be a way to spare the cell reservoir (3, 6). It has been demonstrated that HPgV-1 can be found in T, B, NK cells and monocytes (3), although it is unclear if the primary target cell is a stem cell or differentiated cells. HPgV-1 viremia is found in 1-5% of healthy blood donors in developed countries (3). Viral load can reach 10^7^ copies/ml and transmission through blood transfusion is well-described (7).

Allogeneic hematopoietic stem cell transplantation (allo-HSCT) is a potentially curative treatment for a wide range of haematological malignancies thanks to its graft versus tumor effect (GvT*)* (8). Several immune cell populations are involved in the GvT effect of allo-HSCT, including T and NK cells. NK cells are the first immune effector cell population which reconstitute after allo-HSCT, followed by CD8 T cells and, more lately, by CD4 T cells (9). T cells mainly exert their antitumor effect through alloreactivity against major and minor histocompatibility antigens differing between the donor and the recipients (10). NK cells act through alloreactivity (11) and recognition of activating receptor ligands expressed at the tumor surface (12, 13). Despite these antitumor effects, immune-evasion often occurs after allo-HSCT ultimately resulting in disease relapse. Immune-evasion can take place as a result of mechanisms developed by tumor cells to avoid or actively suppress immune effector cell responses (14). On the other side, inefficient GvT can originate from alterations of immune effector cells after allo-HSCT leading to their reduced cytotoxic activity resulting from their impaired differentiation (15) or from their functional exhaustion (16, 17).

Allo-HSCT recipients’ virome is highly diverse according to the high immune suppression state of this population (18, 19). We and others previously reported a HPgV-1 viremia prevalence rate as high as 42% among allo-HSCT recipients (20). However, no impact of HPgV-1 viremia after allo-HSCT on patients’ outcome has been reported so far. Given the impact of HPgV-1 replication on immune-reconstitution in other contexts of lymphopenia, namely HIV infection (3, 6), we hypothesized that HPgV-1 might affect cellular immune-reconstitution after allo-HSCT. A first exploratory analysis failed to uncover any significant association between HPgV-1 replication and immune reconstitution after allo-HSCT (20). However, the limited sample size of the investigated cohort and several confounders could have masked the potential effect of HPgV-1. To formally investigate the potential impact of HPgV-1 viremia on immune-reconstitution after allo-HSCT, we quantitatively and phenotypically analysed major immune effector cell subsets reconstitution in a cohort of patients displaying HPgV-1 viremia after transplantation and compared them to matched HPgV-1- non-viremic allo-HSCT recipients.

## Methods

### Study design

We selected 40 allo-HSCT recipients from a local cohort (https://clinicaltrials.gov/ct2/show/NCT03642977), including 20 positive for HPgV-1 by rRT-PCR assay on all of their analysed timepoints and 20 negative for HPgV-1 by rRT-PCR assay on all of their analysed time points: among 20 HPgV-1-viremic patients, 20 had rRT-PCR assay performed at day 0/-7, day 30, 3, and 6 months, and 12 had rRT-PCR assay performed at 12 months. Among 20 HPgV-1-non viremic patients, 20 had rRT-PCR assay performed at day 0/-7, day 30, 3, and 6 months, and 15 had rRT-PCR assay performed at 12 months and one patient had rRT-PCR assay performed at 18 months. Patients were matched according to criteria that can influence the immune reconstitution, namely diagnosis, conditioning, graft source, T cell depletion, TBI, donor type and CMV status (**Table 1**). 20 healthy donors from the Geneva University Hospitals blood transfusion center were also analyzed as a control group.

**Table 1:** Clinical characteristics of HPgV-1-non viremic (HPgV-1-) and HPgV-1-viremic (HPgV-1+) allo-HSCT patients.

		HPgV-1 -n = 20	HPgV-1 + n = 20	p value
Age at Tx, median (IQR)		56 (50-63)	56 (51-67)	0.9947
Sex, *n* (%)	Female	10 (50)	6 (30)	0.3332
	male	10	14	
Diagnosis, n (%)	AML	13 (65)	13 (65)	>0.9999
	ALL	4 (20)	4 (20)	
	MDS/MPN	3 (15)	3 (15)	
Graft source, n (%)	PBSC	18 (90)	16 (80)	0.6614
	BM	2 (10)	4 (20)	
TBI, n (%)	Yes	10 (50)	10 (50)	>0.99999
	no	10	10	
Conditioning, n (%)	RIC	11 (55)	10 (50)	>0.99999
	MAC	9 (45)	10 (50)	
Donor type, n (%)	SIB	4 (20)	3 (15)	0.9684
	MUD	11 (55)	11 (55)	
	HAPLO	4 (20)	5 (25)	
	MMUD	1 (5)	1 (5)	
T depletion, n (%)	None	9 (45)	9 (45)	0.9402
	ATG	6 (30)	6 (30)	
	pTCD	2 (10)	3 (15)	
	ATG pTCD	3 (15)	2 (10)	
CMV status	CMV+	14 (70)	11 (55)	0.5145
	CMV-/-	6 (30)	9 (45)	

HPgV-1, Human Pegivirus-1; Tx, transplantation; IQR, interquartile range; n, number; TBI, total body irradiation; CMV, cytomegalovirus; D, donor; R, recipient; AML, acute myeloid leukemia; ALL, acute lymphoid leukemia; MDS/MPN, myelodysplasic syndrome/myeloproliferative neoplasms; PBSC, peripheral blood stem cell; BM, bone marrow; RIC, reduced intensity conditioning; MAC, myeloablative conditioning; SIB, sibling; MUD, matched unrelated donor; HAPLO, haplo identical donor; MMUD, mismatched unrelated donor; ATG, anti-thymoglobulin; pTCD, partial T-cell depletion.

The study was approved by the cantonal ethic committee (CCER 2019-01153) and patients and healthy controls (HC) gave their written informed consent.

### HPgV-1 rRT-PCR assay

190 μl of plasma were spiked with 10 μl of standardized canine distemper virus of known concentration and extracted with the NucliSENS easyMAG (bioMérieux, Geneva, Switzerland) nucleic acid kit in a 25 ul elution volume, according to the manufacturer’s instruction. Extracted RNA was used for HPgV-1-specific rRT-PCR screening analysis, using a previously published assay (21). rRT-PCR assay reaction was performed using the QuantiTect Probe RT-PCR Kit (Qiagen, Valencia, CA. USA) on a StepOnePlus instrument (Applied Biosystems, Rotkreuz, Switzerland) under the following cycling conditions: 50°C for 30 min; 95°C for 15 min; 45 cycles of 15 s at 94°C; and 1 min at 55°C. Data were analysed with the StepOne software V.2 (Applied Biosystems). Analytical sensitivity was assessed with a plasmid-derived transcribed RNA including the target region (kindly provided by Professor JT Stapleton) and showed a lower limit of quantification (LLOQ) = 2.6E3 copies/mL.of plasma.

### Flow cytometry

Peripheral blood mononuclear cells were isolated from EDTA anti-coagulated peripheral blood obtained at 1, 3, 6 and 12 months post-transplantation using Ficoll. Cells were cryopreserved and stored in liquid nitrogen until analysis. After thawing, cryopreserved PBMC were stained with monoclonal antibodies specific for the following antigens: CCR7 (FITC, clone 150503, R&D Systems), CD127 (BV786, clone HIL-7R-M21, BD Biosciences), CD16 (BV480 and PerCPCy5.5, clone 3G8, BD Biosciences), CD25 (APC and PECy7, clone M-A251, BD Biosciences), CD27 (APC-R700, clone M-T271, BD Biosciences), CD27 (BV786, clone L128, BD Biosciences), CD3 (BV711 and PerCPCy5.5, clone UCHT1, BD Biosciences), CD38 (BV605, clone HB7, BD Biosciences), CD4 (BV480, clone SK3, BD Biosciences), CD4 (BV650, clone L200, BD Biosciences), CD45RA (BUV395 and PE, clone HI100, BD Biosciences), CD56 (BV421, clone HCD56, Biolegend), CD57 (BV605, clone QA17A04, Biolegend), CD57 (PECF594, clone NK-1, BD Biosciences), CD8 (BUV737, clone SK1, BD Biosciences), CD8 (BV711, clone RPA-T8, BD Biosciences), HLADR (PECy7, clone L243, BD Biosciences) and PD1 (PE, clone EH12.2H7, Biolegend).

Intracellular staining for cytotoxic molecules and transcription factors was performed overnight at 4°C on fixed and permeabilized cells with FoxP3/transcription factor staining buffer set (e-Bioscience) using antibodies against Eomes (eFluor 660, clone WD1928, eBiosciences), Foxp3 (PECF594, clone 236A/E7, BD Biosciences), GranzymeB (AF700, clone GB11, BD Biosciences), Perforin (FITC, clone B-D48, Diaclone) and Tbet (PE, clone ebio4B10, eBiosciences).

Data were acquired on a BD LSRFortessa Cell Analyzer (BD Biosciences) and analyzed with FlowJo software (FlowJo LLC). Subsets of CD4 and CD8 were defined according to CD45RA, CD27 and CCR7 expression as follows: Naïve T cells (TN) CD45RA+/CD27+, central memory T cells (TCM) CD45RA-/CCR7+, effector memory T cells (TEM) CD45RA-/CCR7- and effector memory re-expressing CD45RA T cells (TEMRA) CD45RA+/CD27- (**Supplemental Figure 1**). Subsets of NK cells were defined based on CD56 and CD16 expression as immature CD56 bright and mature CD56 dim NK cells as shown in **Supplemental Figure 1**.

### Cytokine analysis

Stem cell factor (SCF), IL-7 and IL-15 were quantified in plasma samples using the the LEGENDplex™ Human Hematopoietic Stem Cell Panel (Biolegend). 50 ul of plasma from HSCT patients and healthy controls were diluted 1:1 and the assay was performed according to manufacturer’s instruction. All samples were tested in duplicate. The samples were acquired on a Attune NxT Flow cytometer (Invitrogen) and data were analyzed using the LEGENDplex™ Data Analysis Software.

### Statistical analyses

X^2^ or Fischer exact tests were used for categorical variables. Mann-Whitney test or Wilcoxon matched pairs signed rank test were used for continuous variables. A *P value* <.05 was considered statistically significant. Statistics were performed using R version 3.2.0 and R Studio version 1.3.1056.

## Results

### HPgV-1 replication is associated with higher NK cell absolute numbers at three months after allo-HSCT

**Figure 1:**
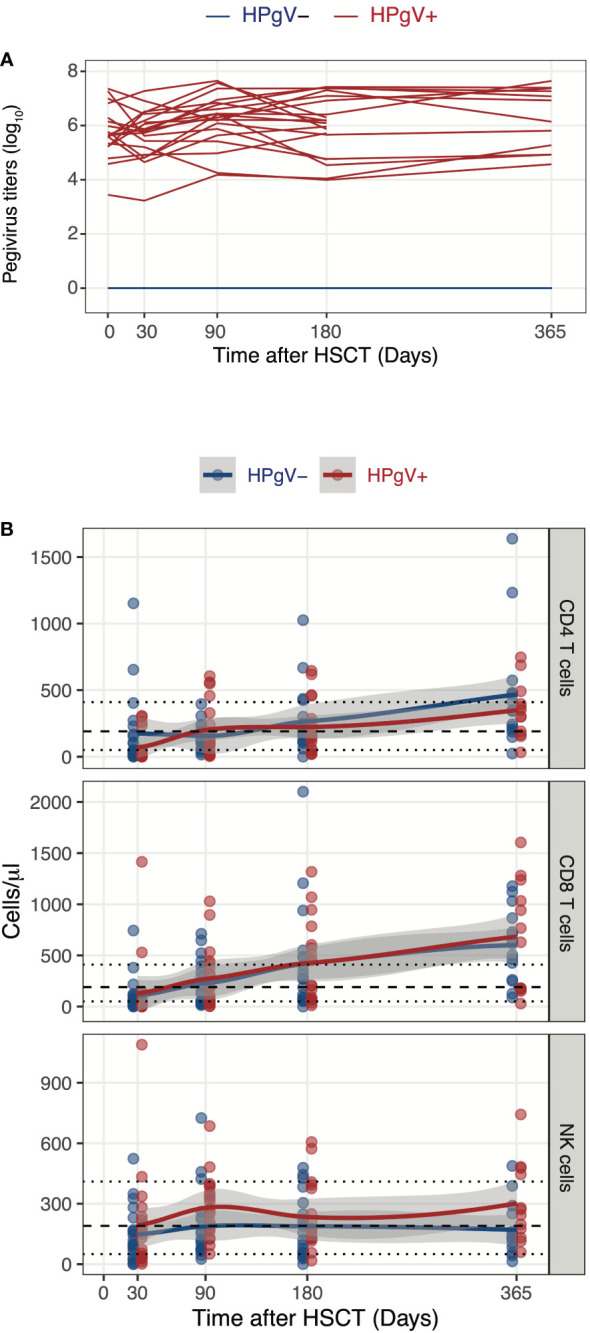
Influence of HPgV-1 replication on immune-reconstitution of mayor lymphocyte subsets after allogeneic HSCT. **(A)** Human Pegivirus-1 titer evolution through one year post-transplantation. Each line represents HPgV-1 titers in log10/RNA copies per ml of plasma in single HSCT recipient. Line colors indicate the patient’ group (red lines: HPgV-1-viremic patients; blue lines: HPgV-1-non viremic patients). Lower limit of quantification (LLOQ) = 2.6E3 copies/mL. **(B)** Absolute CD4 T cell, CD8 T cell and NK cell numbers through one year post-transplantation stratified by patients’ group. Each dot represents one sample, lines represent Loess fit lines and the grey area represents the 95% confidence interval (CI) for the regression fit.

We first assessed the potential impact of HPgV-1 replication on absolute numbers of CD4 and CD8 T cells as well of NK cells at different time-points after allo-HSCT. Patients were selected based on HPgV-1 replication and defined as HPgV-1 positive (HPgV-1+) or negative (HPgV-1-) based on HPgV-1 detection at all or none studied time-points during the first year after transplantation (**Figure 1A**). As expected, the majority of allo-HSCT recipients displayed persistent CD4 T cell lymphopenia up to 1-year post-transplantation while most of them reconstituted normal levels of CD8 T cells and NK cells by 3-months and 1-month post-allo-HSCT respectively (**Figure 1B**). We did not detect any significant differences between HPgV-1+ and HPgV-1- patients in terms of CD4 and CD8 T cell absolute numbers up to 1-year after allo-HSCT (**Figure 1B**, upper and middle panel). In contrast, HPgV-1+ patients displayed significantly higher numbers of NK cells (median 267 (range 51-685) cells/ul) at 3 months after allo-HSCT compared with HPgV-1- patients (119 (26-725) cells/ul, p=0.02; **Figure 1B**, lower panel). Collectively, these data reveal minimal impact of HPgV-1 replication on absolute numbers of major lymphocytes subsets recovered after transplantation with only a slight but significant difference in absolute numbers of NK cells recovered at 3 months after allo-HSCT.

### HPgV-1 replication is associated with further imbalance of CD56^bright^/CD56^dim^ NK cell distribution after allo-HSCT

**Figure 2:**
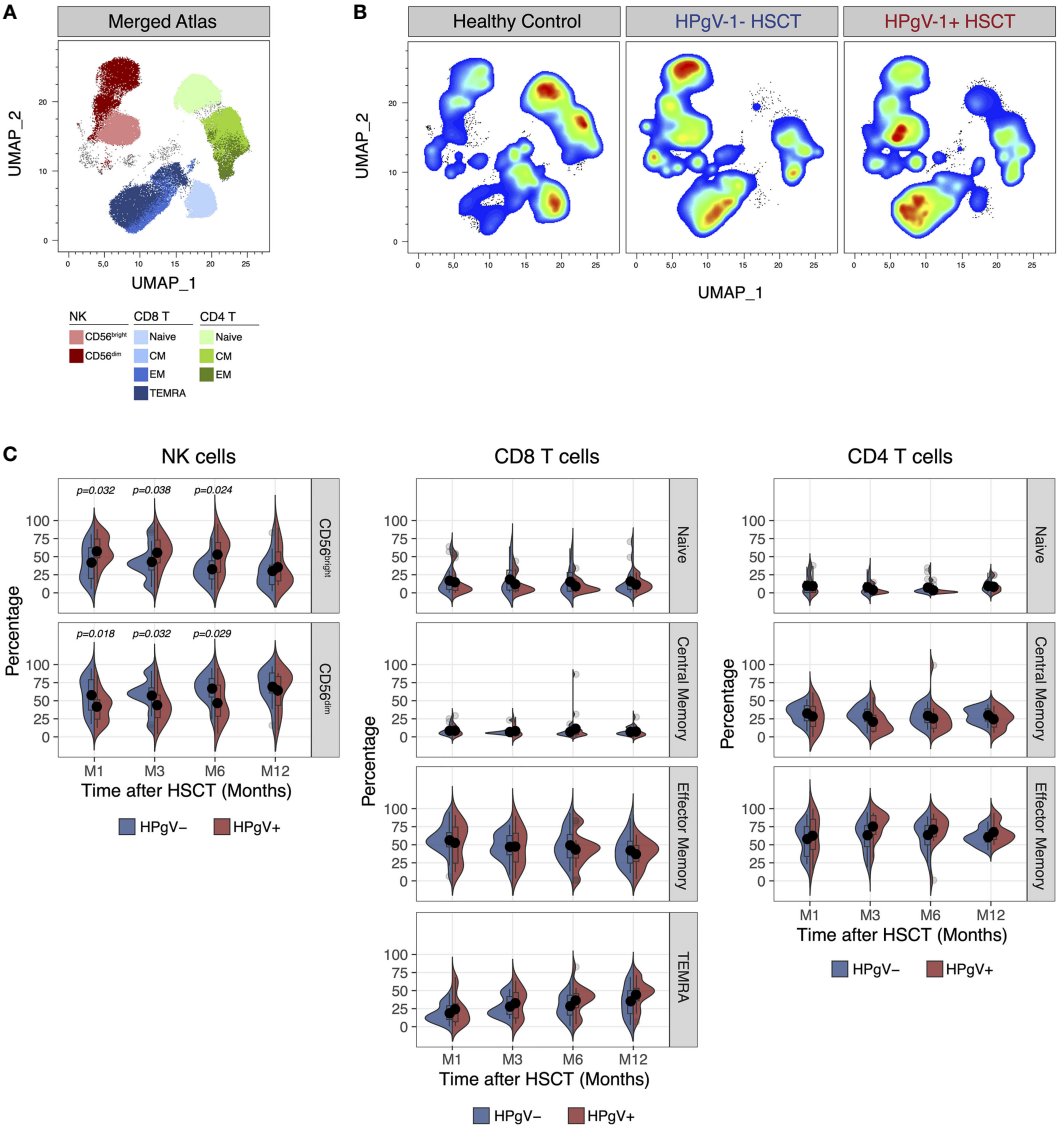
Impact of HPgV-1 viremia on lymphocyte subsets distribution after allogeneic HSCT. **(A, B)** Uniform Manifold Approximation and Projection (UMAP) plots of FACS data obtained from the analysis of live non-B cell lymphocytes recovered from a representative healthy donor, a HPgV-1- and a HPgV-1+ HSCT recipient at 6 months after allogeneic HSCT. Distribution of cell subsets identified by manual gating is shown in the merged atlas **(A)** combining all samples. **(C)** Split-violin plots showing the percentage of the indicated cell subsets among NK (left panels), CD8 (middle panels) and CD4 (right panels) T cells in HPgV-1- (blue violins) and HPgV-1+ (red violins) HSCT recipients. Results in the two patient groups were compared using a nonparametric Mann-Whitney U test.

In addition of quantitative defects, allo-HSCT recipients display major qualitative abnormalities in lymphocyte subsets during immune-reconstitution, namely a skewing in T cell phenotype and repertoire (15) and an immature phenotype in NK cells (22, 23). To evaluate the potential impact of HPgV-1 replication on lymphocyte reconstitution, we next compared the immunophenotype of T and NK cells after transplant in HPgV-1+ and HPgV-1- recipients. Dimensionality reduction of FACS data using Uniform Manifold Approximation and Projection easily identified major T and NK cell subsets in both healthy controls and allo-HSCT recipients (**Figures 2A, B**). As predicted, allo-HSCT recipients displayed a significant increase in less mature CD56^bright^ NK cells and a significant decrease of naïve CD4 and CD8 T cells compared to healthy controls (**Figure 2B**). After stratification of allo-HSCT recipients based on HPgV-1, we detected higher proportions of less differentiated CD56^bright^ NK cells mirrored by lower percentages of mature CD56^dim^ NK cells in HPgV-1+ compared to HPgV-1- allo-HSCT recipients (**Figure 2C**, left panels). Such difference persisted at each time point studied until 6 months after transplantation (**Figure 2C**, left panels). We did not observe any differences in CD8 or CD4 T cell subset distribution (naïve, central memory, effector memory, TEMRA) between HPgV-1+ and HPgV-1- allo-HSCT recipients (**Figure 2C**, middle and right panels).

**Figure 3:**
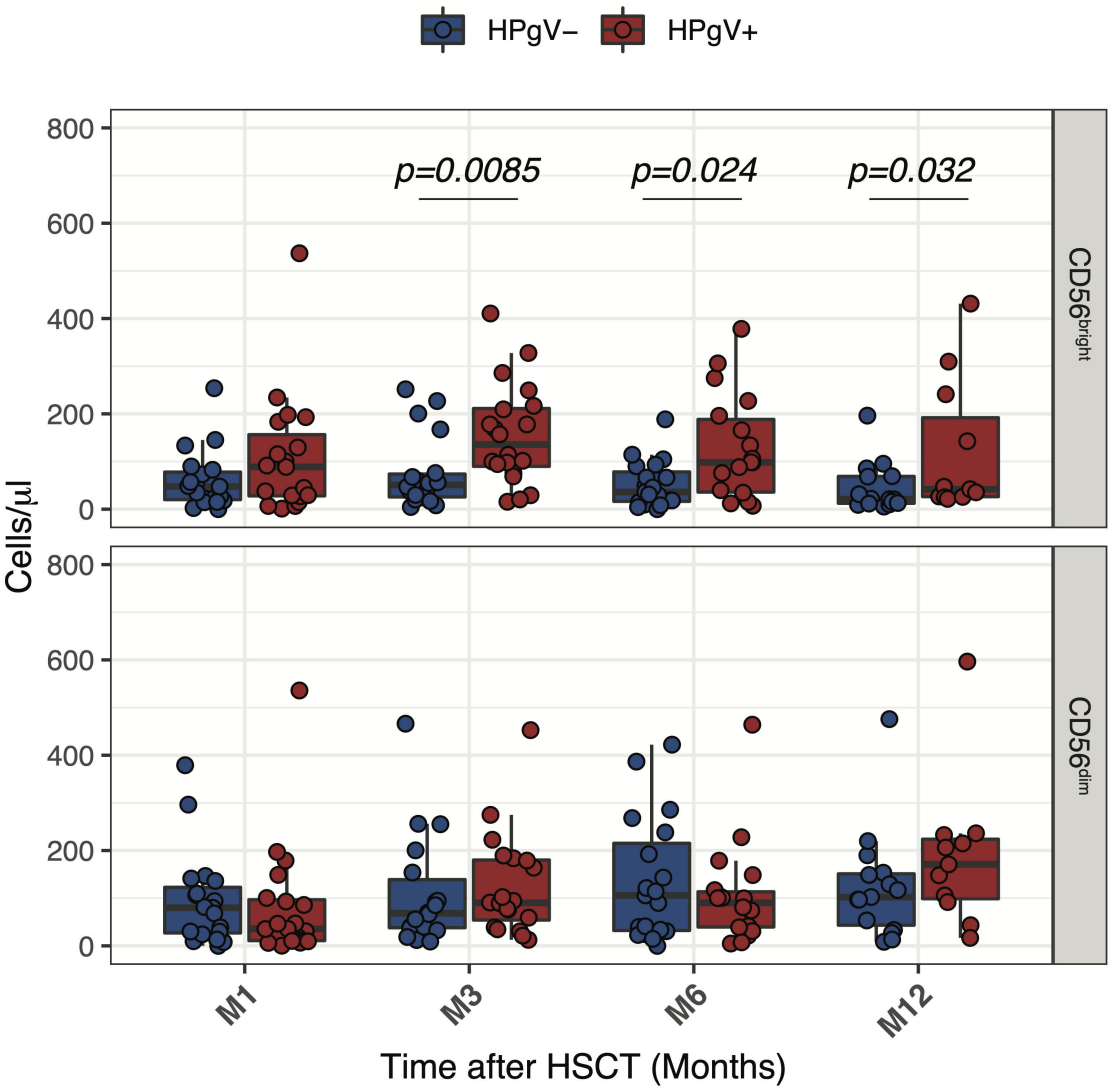
Impact of HPgV-1 viremia on NK cell subsets reconstitution after allogeneic HSCT. Absolute numbers of CD56bright and CD56dim NK cell subsets through one-year post-transplantation in HPgV-1-viremic (HPgV+; red filled symbols) and HPgV-1-non viremic (HPgV-; blue filled symbols) HSCT recipients. Results in the two patient groups were compared using a nonparametric Mann-Whitney U test.

To better assess the relationship between HPgV-1 viremia and the increase of less differentiated CD56^bright^ NK cells, we compared absolute numbers of CD56^bright^ and CD56^dim^ NK cells over the first year of transplantation in HPgV-1+ and HPgV-1- allo-HSCT recipients. As shown in **Figure 3**, this analysis revealed a significant increase in CD56^bright^ NK cell numbers in HPgV-1+ compared to HPgV-1- negative patients at 3, 6 and 12 months after transplantation (**Figure 3**, upper panels) while we did not observe any differences in CD56^dim^ NK cell numbers between the two patient groups.

Collectively, these results indicate that HPgV-1 replication after allo-HSCT was associated with further imbalance of CD56^bright^/CD56^dim^ NK cell distribution due to an increase in CD56^bright^ NK cells during the first year after HSCT in HPgV-1+.

### HPgV-1 replication is associated with higher levels of IL-7 in plasma early after allo-HSCT

**Figure 4:**
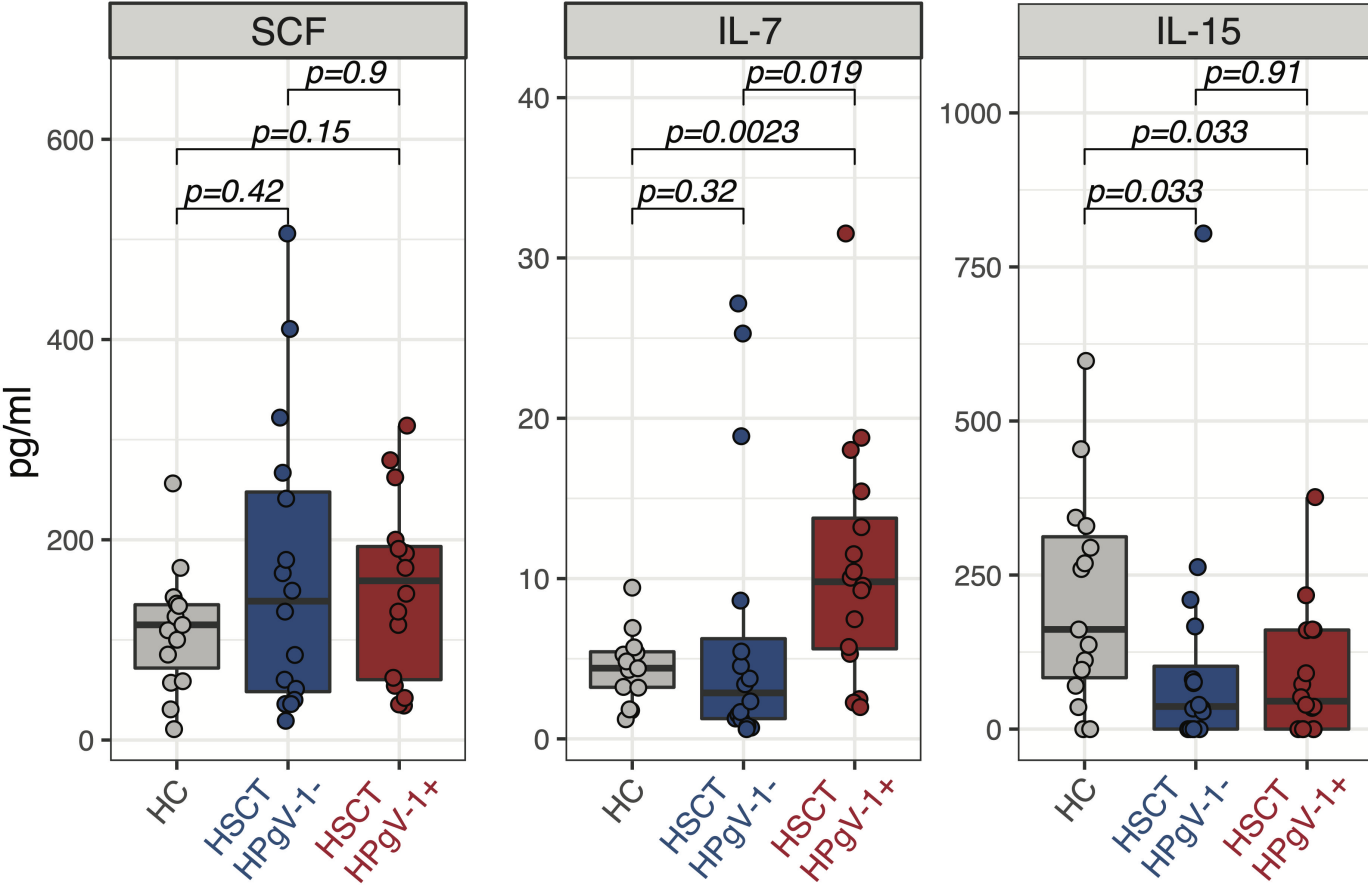
Levels of homeostatic cytokines in HPgV-1-viremic and non-viremic HSCT recipients. Levels of SCF (left panel), IL-7 (middle panel) and IL-15 (right panel) in plasma from HPgV-1-viremic (HPgV+; red filled symbols) and HPgV-1-non viremic (HPgV-; blue filled symbols) HSCT recipients at day 30 after transplantation. Results in the two patient groups were compared using a nonparametric Mann-Whitney U test.

NK cell subsets reconstitution after allo-HSCT is governed by the availability of homeostatic cytokines regulating their proliferation and survival, namely stem cell factor (SCF) and IL-7 for CD56^bright^ NK cells and IL-15 for CD56^dim^ NK cells. We hypothesized that HPgV-1 replication might influence NK cell subsets reconstitution by affecting the levels of homeostatic cytokines. To test this hypothesis, we measured the plasmatic levels of SCF, IL-7 and IL-15 at day 30 after allo-HSCT. No difference was observed in SCF levels between HPgV-1-viremic and non-viremic patients as well as between either patient group and healthy controls (**Figure 4**, left panel). Interestingly, HPgV-1-viremic allo-HSCT recipients displayed significantly higher IL-7 levels compared to both HPgV-1-non-viremic patients and healthy controls (**Figure 4**, middle panel). Both HPgV-1-viremic and HPgV-1-non-viremic allo-HSCT recipients displayed reduced levels of IL-15 compared with healthy controls while we did not detect any significant difference between the two patient groups (**Figure 4**, right panel). Analysis performed at later time points (3, 6 and 12 months) did not detect any significant difference in SCF, IL-7 and IL-15 levels between HPgV-1-viremic and HPgV-1-non-viremic allo-HSCT recipients (data not shown). These data show an association between HPgV-1 replication and IL-7 levels in plasma 30 days after allo-HSCT.

### NK cells from HPgV-1-viremic patients display a less differentiated phenotype after allo-HSCT

**Figure 5:**
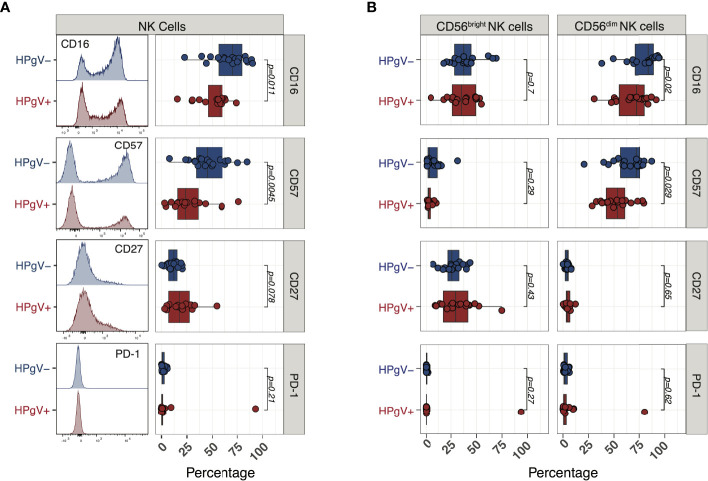
Expression of NK cell differentiation markers in HPgV-1-viremic and non-viremic HSCT recipients. **(A)** Representative FACS histograms (left panels) and summary of percentages (right panels) of expression of the indicated markers in NK cells recovered at 6 months post-HSCT from HPgV-1-viremic (HPgV+; red filled symbols) and HPgV-1-non viremic (HPgV-; blue filled symbols) HSCT recipients. **(B)** Summary of percentages of expression of the indicated markers in CD56bright (left panels) and CD56dim (right panels) NK cells recovered at 6 months post-HSCT from HPgV-1-viremic (HPgV+; red filled symbols) and HPgV-1-non viremic (HPgV-; blue filled symbols) HSCT recipients. Results in the two patient groups were compared using a nonparametric Mann-Whitney U test.

To gain further insights into the phenotypic abnormalities of NK cells during immune-reconstitution after allo-HSCT in the presence of HPgV-1 replication, we next measured the expression of additional markers reflecting NK cell differentiation at 6 months after allo-HSCT. According to their less differentiated phenotype, we observed a significant reduction in the proportion of NK cells expressing the surface Fcγ receptor CD16 in HPgV-1+ patients (58% (16–74)) compared with HPgV-1- individuals (70% (23-90); p=0.011 **Figure 5A**). In addition, NK cells from HPgV-1+ allo-HSCT recipients displayed significantly reduced proportion (24% (3-76) vs 45% (8-85) in HPgV-1- patients; p=0.0045) of cells expressing the N-CAM family molecule CD57, believed to identify cells at final stages of peripheral NK cell maturation (24) (**Figure 5A**). Looking at expression of CD27, a molecule employed to identify less differentiated murine and human NK cells, we observed a trend not reaching statistical significance toward an increase in CD27+ NK cells in HPgV-1+ allo-HSCT recipients 18% (3-55) vs 12% (0.5-21) in HPgV-1- patients; p=0.078; **Figure 5A**). Finally, we analysed the expression of PD-1, a well-established marker of T cell activation and exhaustion whose expression on NK cells is controversial. According to recent reports (25), with the only exception of one patient, we detected minimal PD-1 expression at NK cell surface of either HPgV-1+ or HPgV-1- allo-HSCT recipients (**Figure 5A**). When CD56^bright^ and CD56^dim^ NK cell subsets were analysed separately, the reduction in CD16 and CD56 in HPgV+ recipients was observed in the mature CD56dim NK cell subset while no difference was detected in CD56bright NK cells which, as expected, expressed only low level of these differentiation markers (**Figure 5B**). Collectively, this phenotypic analysis supports an association between HPgV-1 replication and an immature NK cell phenotype during reconstitution after allo-HSCT.

### Impaired NK cell phenotype in HPgV-1-viremic patients is associated with reduced production of the cytotoxic molecule Granzyme B

**Figure 6:**
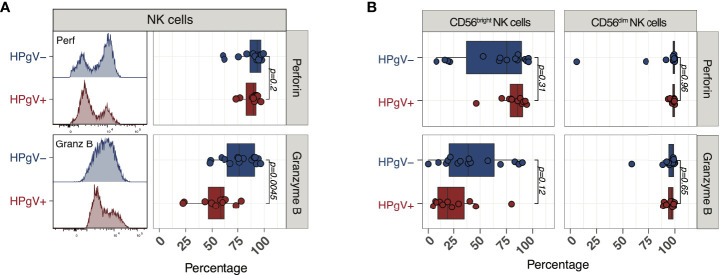
Cytotoxic molecules production in NK cells from HPgV-1-viremic and non-viremic HSCT recipients. **(A)** Representative FACS histograms (left panels) and summary of percentages (right panels) of expression of perforin (upper panels) and granzyme B (lower panels) in NK cells recovered at 6 months post-HSCT from HPgV-1-viremic (HPgV+; red filled symbols) and HPgV-1-non viremic (HPgV-; blue filled symbols) HSCT recipients. **(B)** Summary of percentages of expression of perforin and granzyme B recovered at 6 months post-HSCT from HPgV-1-viremic (HPgV+; red filled symbols) and HPgV-1-non viremic (HPgV-; blue filled symbols) in CD56bright (left panels) and CD56dim (right panels) NK cells from HSCT recipients. Results in the two patient groups were compared using a nonparametric Mann-Whitney U test.

NK cell-mediated cytotoxicity plays a major role in anti-infectious and anti-tumor immunity after allo-HSCT. We hypothesized that the impaired NK cell phenotype observed in HPgV-1+ allo-HSCT recipients might be associated with a reduced capacity to produce cytotoxic molecules. At 6 months after allo-HSCT, we observed no differences in the proportions of perforin producing NK cells between HPgV-1+ and HPgV-1- patients (**Figure 6A**, upper panels). Conversely, we observed a significant reduction in cells expressing the cytotoxic molecule granzyme B among NK cells recovered from HPgV-1+ allo-HSCT recipients (58% (22-78)) compared to cells from HPgV-1- patients (76% (48-97); p=0.0045; **Figure 6A**, lower panels). When CD56^bright^ and CD56^dim^ NK cell subsets were analysed separately, we did not observe any difference in perforin or granzyme B expression in NK cell subsets depending on HPgV-1 replication (**Figure 6B**), indicating that the reduced expression of these molecules detected at the global NK cell population level were mainly due to differences in the CD56^bright^/CD56^dim^ NK cell ratios. Collectively, our analysis reveals that HPgV-1 replication was associated with an immature NK cell phenotype resulting in a decreased expression of the cytotoxic molecule granzyme B.

## Discussion

According to the high prevalence of HPgV-1 among allo-HSCT recipients (26), its role in transplantation outcomes was recently investigated in several studies (26). None of them found any negative impact on clinical outcome, but very few investigated HPgV-1 effects on immune-reconstitution among transplant patients. In this study, we found that HPgV-1-viremic allo-HSCT recipients had a higher NK cell absolute count at 3 months post transplantation, compared to allo-HSCT recipients negative for HPgV-1. More importantly, immunophenotypic analysis of NK cells showed that HPgV-1-viremic patients have a lower proportion of fully differentiated NK cells compared to HPgV-1-non-viremic patients, which was reflected in their reduced expression of granzyme B. This findings corroborate those found in HIV patients (27, 28) and reveal, for the first time, an association between HPgV-1 replication and NK cell differentiation after allo-HSCT.

NK cells are the first immune effector cell subset to fully reconstitute after allo-HSCT (9). For this reason, several groups previously investigated the relationship between NK cell alloreactivity and transplant outcomes, reporting a strong association between NK alloreactivity and disease relapse prevention (11, 29). The ability of NK cells to display alloreactivity without inducing graft-versus-host-disease (30) makes these cells an attractive population for immune effector cellular therapy. Early NK cell reconstitution after allo-HSCT have been associated with improved overall survival as a consequence of reduced relapse rates (31–34). Interestingly, the most robust and long-lasting association with improved outcome was found when CD56^dim^ NK cells were taken into account (33), suggesting that the reconstitution of a fully differentiated, cytotoxic NK cell compartment provides a benefit in terms of disease control. It is well established that NK cells from allo-HSCT recipients display an aberrant phenotype during the first months after transplantation, characterized by an accumulation of less differentiated CD56^bright^ and a decrease in CD56^dim^ NK cells (22, 23, 35). Our data indicate an association between HPgV-1 replication and such aberrant immunophenotype. Moreover, our results pointing toward a delayed reconstitution of mature CD56dim NK cells in HPgV-1-viremic recipients suggest that HPgV-1 might negatively impact patients’ outcome. Our previous report (20) failed to identify such an association but was limited by the great heterogeneity of the patient cohort. Our current study was not designed to test this hypothesis because of the limited number of patients studied. Future larger studies designed to formally address this issue are needed.

The mechanisms by which HPgV-1 affects NK cell reconstitution are unclear. We show here that HPgV-1 replication might influence the production of homeostatic molecules early after transplant, being associated with an increase of IL-7, a cytokine known to favour CD56^bright^ NK cells homeostasis according to their preferential expression of CD127, the alpha chain of IL-7 receptor (35). Conversely, we did not observe any association between HPgV-1 replication and levels of IL-15, the major homeostatic factor for CD56^dim^ NK cells. These findings support a model in which HPgV-1 replication favourably impacts the production of IL-7 early after transplantation thus influencing the homeostatic balance between CD56^bright^ and CD56^dim^ NK cells favouring the first ones. A second, not mutually exclusive, hypothesis is that HPgV-1 might directly modulate NK cell biology. It has been shown that HPgV-1 can infect NK cells (3).We can therefore speculate that, after NK cell infection, HPgV-1 can directly interfere with NK differentiation toward a fully cytotoxic subsets by modulating cellular differentiation processes and/or by inducing a preferential cytopathic effect on fully differentiated NK cells. Future studies will test this second hypotheses.

In agreement with our previous study (20), we found no difference in CD4 and CD8 T cell absolute number and subsets distribution among HPgV-1-viremic and HPgV-1-non-viremic patients within the first year after transplantation. This is in contrast to what observed in other contexts of lymphopenia, namely HIV infection, where an association between HPgV-1 replication and T cell activation and differentiation was found (36–38). One possible explanation is that CD4 and CD8 T cell reconstitution occurs later than NK cells reconstitution after allo-HSCT, at time points when the levels of homeostatic cytokines and the degree of inflammation have progressively normalized in most patients.

Limitations of our study reside in the small sample size and the screening for HPgV-1 that was performed only at predefined timepoint. We can thus not be sure that HPgV-1-viremic patients are constantly viremic throughout the first-year post transplantation, although we selected patients constantly positive at all analysed timepoints, reducing the risk for bias. The size of our patient cohort is too small to allow robust subgroup analyses and future studies are needed to assess the interplay between HPgV-1 and other serostatus and reactivation of other viruses, in particular CMV which plays a major role in NK cell reconstitution after allogeneic HSCT. Also, we did not match patients according to post-transplant events, including immunosuppressive therapy and/or complications (namely graft-versus-host disease), which could have influenced immune reconstitution.

In conclusion, we observed in this study a significant different NK cell, but not T cell, immune-reconstitution after allo-HSCT between HPgV-1-viremic and HPgV-1-non-viremic patients. HPgV-1-viremic patients exhibited a less differentiated NK cell profile compared to HPgV-1-non-viremic patients during the first months after transplantation. Our analysis reveals for the first time an association between the replication of a commensal virus and a well-established phenotypic and functional abnormality of NK cells reconstituting after allo-HSCT. These results stress the importance of understanding the interplay between the human virome and the immune-reconstitution after allo-HSCT.

## Data availability statement

The original contributions presented in the study are included in the article/**Supplementary Material**. Further inquiries can be directed to the corresponding author.

## Ethics statement

The studies involving human participants were reviewed and approved by Geneva cantonal ethics committee. The patients/participants provided their written informed consent to participate in this study.

## Author contributions

AP, FS and D-LV designed the study. AP, SC, AM, SW performed the experiments. AP, M-CZ, A-CM, YC and SM-L collected the clinical data. AP, SC, FS and D-LV interpreted the data. AP and FS analyzed the data, performed statistical analysis, and prepared figures. LK and YC provided essential and critically revised the manuscript. AP, FS, DLV wrote the manuscript. All authors contributed to the article and approved the submitted version.

## Supplementary material

Supplementary Figure 1 Gating strategy for FACS analyses.
