# Erratum: Human pegivirus-1 replication influences NK cell reconstitution after allogeneic haematopoietic stem cell transplantation

**DOI:** 10.3389/fimmu.2023.1170106

**Published:** 2023-03-06

**Authors:** Frontiers Production Office

**Affiliations:** Frontiers Media SA, Lausanne, Switzerland

**Keywords:** human pegivirus-1, NK cell, transplantation, stem cell, CD16, granzyme B, CD57

An omission to the funding section of the original article was made in error. The following sentence has been added: “Open access funding was provided by the University of Geneva”.

The original version of this article has been updated.

